# Involution of breast tissue and mammographic density

**DOI:** 10.1186/s13058-016-0792-3

**Published:** 2016-12-15

**Authors:** Gertraud Maskarinec, Dan Ju, David Horio, Lenora W. M. Loo, Brenda Y. Hernandez

**Affiliations:** University of Hawaii Cancer Center, 701 Ilalo Street, Honolulu, HI 96813 USA

**Keywords:** Breast tissue, Marker expression, Tissue microarray, Immunohistochemistry, Ethnicity

## Abstract

**Background:**

Mammographic density decreases and involution of breast tissue increases with age; both are thought to be risk factors for breast cancer. The current study investigated the relationship between involution or hormone treatment (HT) and breast density among multiethnic patients with breast cancer in Hawaii.

**Methods:**

Patients with breast cancer cases were recruited from a nested case-control study within the Multiethnic Cohort. HT use was self-reported at cohort entry and at the time of the density study. Mammographic density and involution in adjacent non-tumor breast tissue were assessed using established methods. Linear regression was applied to evaluate the correlation between involution and four density measures and to compute adjusted means by involution status while adjusting for confounders.

**Results:**

In the 173 patients with breast cancer, mean percent breast density was 41.2% in mammograms taken approximately 1 year before diagnosis. The respective proportions of women with no, partial, and complete involution were 18.5, 51.4, and 30.1%, respectively and the adjusted density values for these categories were 32.5, 39.2, and 40.2% (*p* = 0.15). In contrast, the size of the dense area was significantly associated with involution (*p* = 0.001); the values ranged from 29.7 cm^2^ for no involution to 48.0 cm^2^ for complete involution. The size of the total breast area but not of the non-dense areas was also larger with progressive involution. Percent density and dense area were significantly higher in women with combined HT use.

**Conclusions:**

Contrary to previous reports, greater lobular involution was not related to lower mammographic density but to higher dense area. Possibly, percent density during the involution process depends on the timing of mammographic density assessment, as epithelial tissue is first replaced with radiographically dense stromal tissue and only later with fat.

## Background

The human breast has 15–20 lobes, each with many lobules containing acini, the secretory structures of the breast [1]. Lobules are surrounded by varying amounts of stroma and fat. With age, the progressive replacement of glandular elements with collagen and fat is accompanied by histologic loss of epithelial cells available for malignant transformation and results in lobules characterized by acinar epithelia and fibrosis of the intralobular stroma [2, 3]. Delayed involution [2], i.e., variations in the rate or extent of decrease in the number and size of breast lobules with aging contributes to breast cancer risk [4]. For example, women with only type I lobules, i.e., complete involution, had a lower breast cancer risk than women with type II and type III lobules [2, 5].

Involution of the glandular structures of the breast may be reflected in mammographic density, an independent predictor of breast cancer. Women with extensive breast density (>75%) have fourfold to six-fold higher risk than women with low (<5%) density [6, 7]. Tissues with low and high mammographic density differ in their proportion of stroma, collagen, epithelium, and fat [8], but dense areas represent epithelial and stromal tissues [9]. Biological processes underlying breast involution and density may share common hormonal influences, such as hormone therapy (HT), which affects breast cancer risk, involution, and mammographic density [10, 11].

In women attending the Mayo clinic with benign breast disease, the mean percent density values by none, partial, and complete involution status were 22, 22, and 17%, respectively, after adjustment for known risk factors [1]. In a case-control study within the same population [6], the combination of no involution plus dense breasts was related to fourfold higher risk of breast cancer. A later report on women with benign biopsies also described a significant inverse association between involution and percent density but not absolute density, particularly among premenopausal women [12]. In the current study, we analyzed the association between breast density and involution as the primary objective, but also examined the well-known association between HT and mammographic density among women in Hawaii.

## Methods

### Study population

The current pathologic investigation [13] is based on Hawaii participants of the Multiethnic Cohort (MEC), who took part in a nested case-control (NCC) study of mammographic density and breast cancer risk [14]. The MEC was established in 1993–1996 by mailing a self-administered, 26-page questionnaire asking about demographic, anthropometric, and medical factors to men and women ages 45–75 years residing in Hawaii and California [15]. Additional information on HT, menopausal status, and mammograms was obtained when women enrolled in the NCC study.

The MEC is linked annually to the statewide Hawaii Tumor Registry (HTR) to identify incident cancer cases. Invitations for the pathology study were mailed to 430 of the 607 women in the NCC study, for whom tumor blocks were available through the HTR. Of the 279 women with breast tumor tissue represented on microarrays [16], blocks with sufficient non-tumor tissue to assess breast involution were only available for 173 women, as many of the biopsy specimens were small and did not contain enough benign tissue to evaluate involution. The Institutional Review Board at the University of Hawaii approved all study protocols; all women signed informed consent to be part of the NCC and the pathology investigation.

### Mammographic density assessment

**Fig. 1:**
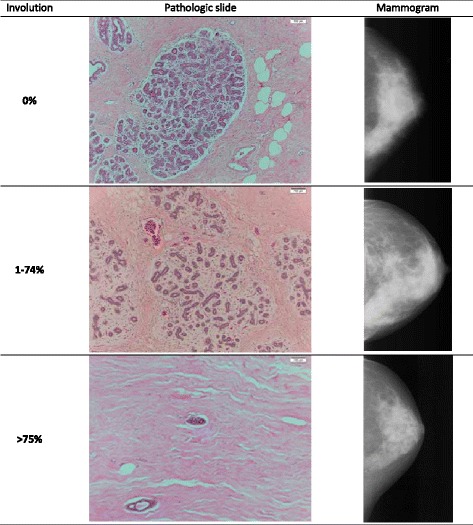
Examples of involution status (0%, 1–74%, and ≥75%) and mammographic density

Mammographic images of study participants from clinics throughout the State of Hawaii were retrieved and digitized using a Kodak LS 85 Film Digitizer (Kodak, Rochester, NY, USA) with a pixel size of 260 μm [14]. For the pathology study, the results of craniocaudal-projection images obtained closest to, but before, the date of diagnosis were selected [13]. Using the Cumulus software developed at the University of Toronto, Canada [17], the scanned images for both breasts were assessed for density (Fig. 1) by one reader (GM) who was blinded to case status and time sequence of mammograms [14]. Percent density was computed as the dense area divided by the total breast area and non-dense area as the difference between the total breast and the dense area. The intraclass correlation coefficients derived from duplicate readings were 0.96 for the size of the dense area and 0.97 for percent density [14].

### Pathologic assessment of lobular involution

Pathologic blocks were retrieved through the tissue repository of the HTR. H&E slides (mean = 2.6 per woman) of normal (non-tumor) tissue adjacent to but not immediately bordering the tumor were prepared. The extent of breast tissue lobular involution (Fig. 1) in breast tissue was evaluated by a pathologist (DH) who was blinded to mammographic, clinical, and risk factor data. The extent of lobular involution was based on morphologic assessment of terminal duct lobular units (TDLUs) classified into one of three categories [18]: no involution (0% involuted), partial (1–74%), or complete (≥75%). TDLUs with abnormal features (e.g. dilation of acini, hyperplasia, metaplasia, or calcifications) were excluded from evaluation.

### Statistical analysis

The statistical analysis was performed using the SAS statistical software (SAS Institute Inc., Cary, NC, USA). We computed frequencies and means for variables of interest by involution status. Multiple linear regression was applied to evaluate the relationship between involution and mammographic measures (percent density, dense area, non-dense area, and total area) and to compute adjusted means by the category of involution and HT. Separate models for premenopausal and postmenopausal women were analyzed. Trend tests were performed using the indicator variables for involution status as a continuous variable. All models were adjusted for covariates known to be associated with breast density: age at mammogram, body mass index (BMI) (<25, 25 to <30, ≥30 kg/m^2^), ethnicity (Caucasian, Native Hawaiian, Japanese, other), parity (0, 1–2, 3+), menopausal status, and HT use (none, estrogen only, estrogen plus progesterone).

## Results

The mean age at breast cancer diagnosis in the 173 women in the involution study (62% of the original study population) was 59.7 ± 8.0 years as compared to 61.4 ± 8.7 years in the 279 participants in the original study (Table 1). The distributions of ethnicity, BMI, and reproductive factors, HT use, and cancer stage) were very similar in the subset and the original study, but percent density and dense area were slightly higher and non-dense and total area lower than in the entire study population, in mammograms taken 1.1 ± 1.1 years before diagnosis. Approximately half of the women (51.4%) were classified as having partial involution, followed by 30.1% and 18.5% with complete and no involution, respectively. The majority of women were postmenopausal (69.4%). The population represented Caucasians (33.5%), Japanese (46.2%), Native Hawaiians (11.0%), and other ethnic groups (9.3%). Past or present HT use was reported by 65.9% of all women. The cancer stage in the majority of women was localized (60.7%), followed by *in situ* (22.5%) and advanced (12.1%). Involution status differed significantly by age at diagnosis (*p* = 0.01) with low involution among women under 50 years of age and higher proportions in women 60 years and older (Table 1). Partial and complete involution did not differ significantly by previous HT (*p* = 0.58).

**Table 1:** Characteristics of 173 study participants with breast cancer in the Multiethnic Cohort

Characteristic^a^	Original study	Involution study	No involution	Partial involution	Complete involution	*P* value^b^
			(0%)	(1–74%)	(≥75%)	
Sample size (%)	279	173	32 (18.5)	89 (51.4)	52 (30.1)	N/A
Age at diagnosis						
< 50 years	24 (8.6)	15 (8.7)	7 (21.9)	7 (7.9)	1 (1.9)	0.01
50 to <55 years	56 (20.1)	42 (24.3)	6 (18.8)	25 (28.1)	11 (21.2)	
55 to <60 years	55 (19.7)	40 (23.1)	12 (37.5)	18 (20.2)	10 (19.2)	
60 to <65 years	44 (14.3)	31 (17.9)	3 (9.4)	17 (19.1)	11 (21.2)	
65+ years	104 (37.3)	45 (26.0)	4 (12.5)	22 (24.7)	19 (36.5)	
Menopausal status (%)						
Premenopausal	76 (27.2)	53 (30.6)	12 (37.5)	28 (31.5)	13 (25.0)	0.47
Postmenopausal	203 (72.8)	120 (69.4)	20 (62.5)	61 (68.5)	39 (75.0)	
Ethnicity (%)						
Caucasian	97 (34.8)	58 (33.5)	7 (21.9)	35 (39.3)	16 (30.8)	0.39
Japanese American	121 (43.4)	80 (46.2)	16 (50.0)	39 (43.8)	25 (48.1)	
Native Hawaiian	36 (12.9)	19 (11.0)	6 (18.8)	6 (6.7)	7 (13.5)	
Other	25 (8.9)	16 (9.3)	3 (9.4)	9 (7.7)	4 (7.7)	
Parity						
Nulliparous	47 (16.9)	25 (14.4)	6 (18.8)	8 (9.0)	11 (21.2)	0.16
1–2 children	110 (39.4)	69 (39.9)	15(46.9)	38 (42.7)	16 (30.8)	
3 or more children	122 (43.7)	79 (45.7)	11 (34.4)	43 (48.3)	25 (46.1)	
Hormone use (%)						
Never	102 (36.5)	59 (34.1)	9 (28.1)	28 (31.5)	22 (42.3)	0.58
Estrogen	97 (34.8)	62 (35.8)	11 (34.4)	34 (38.2)	17 (32.7)	
Estrogen/progesterone	80 (28.7)	52 (30.1)	12 (37.5)	27 (30.3)	13 (25.0)	
Body mass index						
< 25 kg/m^2^	167 (59.9)	104 (60.1)	18 (56.3)	56 (62.9)	30 (57.7)	0.60
25 to <30 kg/m^2^	81 (29.0)	54 (31.2)	12 (37.5)	27 (30.3)	17 (28.9)	
30+ kg/m^2^	31 (11.1)	15 (8.7)	2 (6.3)	6 (6.7)	7 (13.5)	
Tumor stage (%)						
*In situ*	61 (21.9)	39 (22.6)	10 (31.3)	18 (20.2)	11 (21.2)	0.60
Localized	169 (60.6)	105 (60.7)	20 (62.5)	53 (59.6)	32 (61.5)	
Advanced	37 (13.2)	21 (12.1)	2 (6.3)	13 (14.6)	6 (11.5)	
Missing	12 (4.3)	8 (4.6)	0 (0.0)	5 (5.6)	3 (5.8)	
Mammographic measures						
Percent density	37.6	41.2	38.8	42.8	40.0	0.96
Dense area (cm^2^)	37.8	40.8	32.6	41.6	44.7	0.04
Non-dense area (cm^2^)	77.5	67.8	63.9	64.9	75.1	0.24
Total area (cm^2^)	115.3	108.6	96.5	106.4	119.5	0.02

^a^Numbers and percentages except for mammographic measures; percentages may not add up to 100 due to rounding

^b^Based on the chi-square test for categorical variables and general linear models for continuous variables

**Fig. 2:**
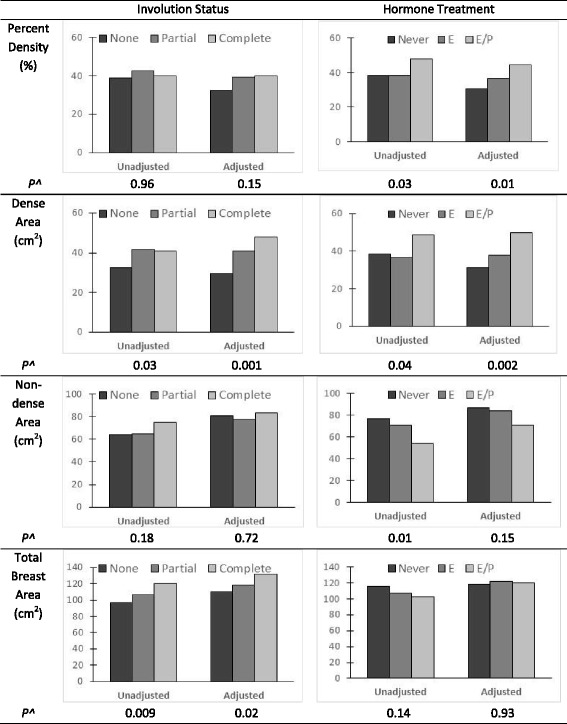
Mammographic measure by involution status and hormone treatment. Means and *p* values were obtained using general linear models and were adjusted for ethnicity, age, body mass index, menopausal status, and parity. *E* estrogen, *P* progesterone

The mean breast density in all women was 41.2%; the unadjusted values (Fig. 2) were similar for no, partial, and complete involution (38.8, 42.8, and 40.0%, respectively; *p* = 0.96). The difference across categories increased after adjustment for confounders (32.5, 39.2, and 40.2%, respectively) but was not significant (*p* = 0.15). In contrast, the size of the dense area differed significantly in the unadjusted (*p* = 0.03) and adjusted models (*p* = 0.001) with the highest value for women with complete involution (48.0 cm^2^) and the lowest (29.7 cm^2^) for those with no involution. The non-dense area varied little (80.9, 77.5, and 83.4 cm^2^, respectively; *p* = 0.72), whereas the total breast area was significantly higher with more advanced involution before (*p* = 0.009) and after adjustment (110.6, 118.4, 131.4 cm^2^, respectively, *p* = 0.02). Interaction terms and stratified analyses did not indicate any differences by menopausal status. Involution was not associated with percent density in premenopausal or postmenopausal women (*p* = 0.27 and 0.42, respectively), whereas the dense area remained higher with progressive involution (*p* = 0.04 and 0.02, respectively).

HT was significantly associated with several mammographic measures (Fig. 2). Percent density (30.7, 36.5, and 44.6%; *p* = 0.01) and dense area (31.2, 37.7, 49.7 cm^2^; *p* = 0.002) were higher in women with combined HT use, but the non-dense and the total area was not associated with HT use.

## Discussion

As in previous reports, age was a strong predictor of involution among MEC participants in Hawaii; complete involution is uncommon before the age of 50 years and increases to as much as 50% in women aged 60 years and older [2, 9]. In the current analysis, women with more advanced involution had greater dense and total breast areas, whereas percent density and non-dense area were not related to involution. This lack of an association between involution and percent density is in contrast to a Mayo study, in which there was lower percent density with more advanced involution [1], and an investigation of benign diagnostic biopsies reporting a direct association between percent mammographic density and TDLU count around the biopsy site, but primarily in premenopausal women [12]. There was no association between involution and absolute dense area/volume in either of these reports. Unlike in the study by Gierach et al. [12], the associations in the current analysis did not differ by menopausal status. In a study from Vermont that examined the relationship between circulating IGF-1 and involution or breast density, the positive correlation between TDLU counts and percent density was not significant after adjustment for confounders [19].

Our finding of larger dense areas on mammograms of more involuted breasts suggests that women with a larger degree of involution may not have more fatty tissue but rather a higher proportion of stromal tissue. Thus, the mammographic images appear radiographically dense despite being poor in epithelial cells. In the two previous reports [1, 12] of inverse associations between involution and percent density, but not with dense area, the authors argued that lobules were not replaced exclusively by stroma but by a combination of stroma and fat [1]. Possible reasons for the discrepant results include the small sample size of 173 women, the ethnic diversity, and the fact that all women were diagnosed with breast cancer. As the pathology specimens were obtained from the area surrounding the tumor in previous studies, they likely represented higher-risk tissue than breast tissue identified as benign on biopsy obtained prior to cancer diagnosis [1, 12].

Our findings confirm the strong association between mammographic density and HT, especially estrogen and progesterone combined [20], but the degree of involution was only weakly associated with HT in the current analysis, although previous reports describe less involution in women taking HT and suggest that estrogens play a role in involution and breast density [1]. It has also been reported that postmenopausal women with higher estradiol levels were more likely to have higher TDLU counts [21]. While exogenous intake of estrogen may stimulate growth in breast tissue and provide more epithelial cells at risk of mutation, estrogen could also delay the age-related involution of the lobules through mechanisms that are yet to be defined [4]. The importance of breast tissue structure was emphasized by a case-control study within the Nurses’ Health study [5]; women with predominant type I and no type III lobules had a 30% lower risk of breast cancer than those with no type I lobules or mixed lobule types.

Strengths of the current study include the ethnic diversity with a wide range of mammographic density values [14], the availability of many covariates, and the quantitative breast density assessment providing dense and non-dense area measures. However, the major weakness is that all participants of the current study were diagnosed with breast cancer, thus, their breast tissue may have been less involuted than tissue in women with benign biopsies [1, 12] and may be responsible for the observed positive associations. Other limitations include the lack of slides for more than 100 women who participated in the original study [16]. Although the distribution by stage of diagnosis for the current study samples was similar to the original 279 study participants (Table 1), the study population was slightly younger and the total breast area was larger and the dense area smaller, resulting in lower percent density (Table 1). As different approaches in assessing involution have been applied, i.e., TDLU counts, TDLU span, and acini counts/TDLU, it is not clear how comparable our findings, based on a simple method, are to previous reports. Nevertheless, all measures started declining significantly in the third decade of age and all metrics were statistically significantly lower among postmenopausal women [18].

## Conclusions

Contrary to previous reports [1, 12], greater lobular involution was not related to lower mammographic density but to higher dense area, in the current analysis of a small number of breast cancer cases. It is possible that percent density during the involution process depends on the timing of mammographic density assessment, as epithelial tissue is first replaced with radiographically dense stromal tissue and only later with fat. Hence, percent density in breast tissue undergoing involution may depend on the timing within the involution process when mammographic density was assessed. In addition to epithelial cell death and remodeling, the presence of immune cells and inflammatory response to remove debris may influence the appearance of the breast [3, 22]. Based on the limited evidence, it is tempting to consider involution and mammographic density as intermediate endpoints in breast carcinogenesis [6], as both measures are related to aging, as collagen, glandular area, and nuclear area [10], and rely on visual assessments of tissue architecture, one at the microscopic and the other at the macroscopic level. However, at this time we do not have information on the average rate of involution, the timing of the transitional process, and possible differences in the process among women with and without breast cancer.

## Abbreviations

BMI: body mass index
H&E: hematoxylin and eosin
HT: hormone use
HTR: Hawaii Tumor Registry
MEC: multiethnic cohort
NCC: nested case-control study
TDLU: terminal duct lobular unit
